# Addressing barriers and facilitators to family planning uptake among adolescent girls and young women in the anticipated context of long-acting antiretroviral therapy (LA ART)

**DOI:** 10.1371/journal.pgph.0007546

**Published:** 2026-11-10

**Authors:** Shukri Ahmed Hassan, Whitney Biegon, Mehar Maju, Dennis Munyoro, Salim Bakari, Eunice Kaguiri, Anjellah Jumah, Mark Omollo, Valerie Obare, Edith Apondi, Edwin Were, Caitlin Bernard, Rena C. Patel

**Affiliations:** 1 Department of Epidemiology, Columbia University, New York, New York, United States of America; 2 Academic Model Providing Access to Healthcare (AMPATH), Eldoret, Kenya; 3 Department of Medicine, University of Washington, Seattle, Washington, United States of America; 4 Ministry of Health, National AIDS and STI Control program (NASCOP), Nairobi, Kenya; 5 Department of Reproductive Health, Moi University College of Health Sciences, Eldoret, Kenya; 6 Department of Obstetrics and Gynecology, Indiana University School of Medicine, Indianapolis, Indiana, United States of America; 7 Department of Medicine, University of Alabama Birmingham, Birmingham, Alabama, United States of America; Washington University in St. Louis, UNITED STATES OF AMERICA

## Abstract

Adolescent girls and young women (AGYW) aged 15–24 years bear a disproportionate HIV burden globally, with nearly twice as many AGYW living with HIV as adolescent boys and young men. Despite a 63% reduction in new infections since 2000, 210,000 new infections occur annually among AGYW, four times higher than the 2025 global target of 50,000. Despite emerging opportunities for integrated service delivery, including now through long-acting antiretroviral therapy (LA ART), significant gaps remain in understanding barriers and facilitators to family planning (FP) access among AGYW living with HIV from adolescent and stakeholder perspectives. We conducted focus group discussions (FGDs) with four stakeholder groups in western Kenya (November 2021–April 2022): adolescents and young people living with HIV (AYPLHIV), healthcare providers, advocates, and policymakers. Participants were recruited through purposive and snowball sampling from Rafiki Clinic at Moi Teaching and Referral Hospital in Eldoret, Kenya, and transcripts were analyzed using inductive and deductive approaches organized by the socioecological model (SEM). Seven FGDs were conducted with 58 participants total. Four themes emerged across SEM levels: at the policy level, parental consent requirements created policy-level implementation challenges; at the health systems level, barriers included provider stigma, inadequate youth-friendly services, and supply chain gaps; at the interpersonal level, peer mentors were trusted but could perpetuate misinformation, parental disapproval persisted, and male partner involvement was variable, and at the individual level, adolescents preferred discreet, low-maintenance methods, though myths, side effect fears, and procedural anxiety influenced choices. Effective interventions require comprehensive approaches combining policy clarification, provider training, service integration, male partner and parental engagement, evidence-based peer mentor programs, and removal of cost barriers to long-acting reversible contraceptives. Integration of FP within LA ART delivery platforms offers promising opportunities to normalize contraceptive use and improve reproductive health outcomes for AGYW living with HIV.

## Introduction

Adolescent girls and young women (AGYW) aged 15–24 years bear a disproportionate burden of HIV infection globally, representing a critical population at the intersection of gender inequality and public health vulnerability [1,2]. In 2023, an estimated 1.9 million AGYW were living with HIV, compared to 1.2 million adolescent boys and young men [1–3]. Although global new infections fell by 63% between 2000 and 2023, approximately 210,000 new infections still occurred among AGYW that year, more than four times the 2025 global target of 50,000 [1–4]. The burden remains concentrated in sub-Saharan Africa, where 77% of AGYW who acquired HIV in 2023 reside [1–4]. In this region, HIV prevalence among AGYW is more than three times higher than their male counterparts [1–4]. These disparities reflect longstanding structural factors including gender inequities, limited access to sexual and reproductive health (SRH) services, power imbalances in sexual relationships, and exposure to gender-based violence, all of which increase HIV risk while simultaneously creating barriers to prevention, testing, and treatment services [1–4]. The dual burden of unplanned pregnancy and HIV/sexually transmitted infections (STIs) further compounds the vulnerability of AGYW while the lagging disparities in treatment adherence and viral suppression rates compared to older women and adult populations living with HIV underscore the urgent need for innovative, youth-friendly interventions that address both biomedical and structural determinants of HIV acquisition and care engagement [5,6].

Unplanned pregnancies among AGYW create cascading effects that intersect dangerously with HIV vulnerability [6–8]. Educational disruption and economic hardship frequently follow, reducing their autonomy and decision-making power in relationships [9]. Adolescent pregnancies carry significantly increased risks of maternal morbidity and mortality, and social stigma can lead to family rejection and community exclusion [10]. Limited access to effective contraception may drive young women into exploitative relationships or high-risk sexual behaviors, creating particularly dangerous intersections between unmet contraceptive needs and HIV risks [11–13]. Fear of seeking care following unsafe abortions, driven by legal concerns and stigma, can delay treatment and increase risks, highlighting the urgent need for integrated approaches that address both reproductive health and HIV prevention simultaneously [12–15]. The same structural barriers compound HIV treatment challenges; economic instability limits healthcare access, educational disruption interferes with medication adherence, and social stigma can lead to disengagement from HIV services [16,17]. Yet, the evolving landscape of HIV treatment presents unprecedented opportunities through innovative service delivery models, particularly as long-acting antiretroviral therapy (LA ART) creates new paradigms for healthcare engagement [18–20]. LA ART delivery can serve as routine contact points for AGYW living with HIV (AGYWLHIV), offering natural opportunities for integrated models that combine family planning (FP) with HIV prevention and treatment. This integration has been shown to increase uptake and continuity of both services, reduce missed opportunities for comprehensive care, and be more cost-effective than standalone programs [21].

Despite growing evidence on barriers to FP among AGYW in sub-Saharan Africa, limited research has examined how the anticipated introduction of LA ART may reshape these dynamics, particularly from multi-stakeholder perspectives [21,22]. This study addresses that gap by identifying barriers and facilitators to FP access from the perspectives of adolescents, advocates, healthcare providers, and policymakers, and examining how these factors intersect with opportunities for FP integration within HIV prevention and treatment platforms. To capture the multi-level nature of these influences, we apply the socioecological model (SEM), a framework that organizes determinants of health behavior across individual, interpersonal, health system, and policy levels. The SEM is well suited to this context, as FP access among AGYWLHIV is shaped by factors operating simultaneously across these levels. By centering adolescent voices alongside insights from key stakeholders, this research generates actionable evidence to guide the design of responsive, youth-friendly, and integrated reproductive health services that leverage emerging opportunities in HIV care delivery.

## Methods

### Ethics statement

This study was approved by the Institutional Research and Ethics Committee (IREC) at Moi University, Eldoret, Kenya (0003912) and the University of Washington Institutional Review Board, Seattle, United States of America (IRB ID STUDY00012640). Verbal informed consent was obtained from all adult participants, including healthcare providers, advocates, and policymakers. For participants under 18 years of age, verbal informed consent was obtained from a parent or guardian, and verbal assent was obtained from the adolescent. Details regarding the study setting, team composition, and positionality have been previously published [24,25].

### Study setting, study team, and positionality

This qualitative study was conducted at Moi Teaching and Referral Hospital (MTRH) in Eldoret, western Kenya, between November 2021 and April 2022. The study was conducted at Rafiki Clinic, the only youth-friendly, integrated HIV treatment and family planning clinic in Uasin Gishu County, established in 2016. Rafiki Clinic provides comprehensive services including HIV care, family planning, STI screening, and mental health services, and also conducts outreach targeting street-connected youth, orphans, and vulnerable children. A youth advisory board, compromising of peer mentors and youth living with HIV, guided the study’s design, the consenting process, and the design and selection of a study logo. The study team comprised of academic researchers, study staff, and youth peer educators, most of whom were Black Kenyans except for four individuals (two South Asian Americans, one white American, and one Somali American). Throughout all phases of the project, the team regularly met and generally utilized a consensus-driven approach for decision-making. Non-Kenyan team members collaborated closely with Kenyan counterparts to ensure that results and interpretations aligned with the perspectives, beliefs, and lived experiences of the Kenyan team members. We acknowledge that the diverse positionalities of team members, across nationality, professional role, and lived experience, may have shaped interpretive decisions, and the consensus-driven process was designed to surface and navigate these differences transparently. We utilized the Consolidated criteria for Reporting Qualitative research (COREQ) [23], as a reporting guide.

### Data collection

We conducted focus group discussions (FGDs) with four stakeholder groups: (1) both male and female AYPLHIV, (2) healthcare providers, (3) advocates, and (4) policymakers. We aimed to conduct two FGDs per group; however, only one FGD was conducted with policymakers due to scheduling conflicts and limited availability of participants at both the national and county levels. Recruitment took place from 26/11/2021 to 05/04/2022. Participants were recruited through purposive and snowball sampling guided by the study team and youth peer mentors. AYPLHIV were identified and recruited from Rafiki Clinic during scheduled appointments by research staff, using flyers and word-of-mouth to identify an initial pool of potential participants, who were then asked to recommend 1–2 additional participants. Healthcare providers were recruited through facility leadership, identifying potential participants from Rafiki Clinic and other HIV and family planning clinics at MTRH, all of whom were directly involved in HIV care. Advocates and policymakers were identified through key organizations and professional networks, who were contacted to provide representatives willing to participate and to recommend 1–2 additional groups or individuals for inclusion. Advocates were recruited from non-profit organizations working in the areas of HIV, youth health, and family planning, reflecting community-based settings focused on these issues.

The AYPLHIV FGDs included both male and female participants and were not stratified by sex or age. Among the 14 AYPLHIV participants, half were female and half were male, and half were under 18 years old. Mixed-gender FGDs were a deliberate design choice to capture a range of perspectives within this group. Male AYPLHIV were included to capture the perspectives of male peers and partners, who shape the interpersonal context of AGYW’s contraceptive decision-making.

Prior to each FGD, research staff obtained oral informed consent from all participants in their preferred language, either English or Kiswahili. For participants who were minors, assent was obtained from the participant and consent was obtained from a parent or guardian prior to participation. Verbal consent was documented by research staff at the time of the session. All FGDs were audio-recorded, (conducted by facilitators already working at MTRH who were chosen based on their experience conducting FGDs and working with AYPLHIV) transcribed, and translated into English by the Qualitative Research Core at the study site. Translated transcripts were reviewed against the original audio recordings by the FGD facilitator for the relevant session to verify translation fidelity, and discrepancies were resolved through discussion with the Qualitative Research Core. The Qualitative Research Core consists of trained research assistants with expertise in qualitative data collection, transcription, and translation. The FGD guides, informed by existing literature and the SEM [26], explored four main domains: living with HIV, perspectives on FP, perceived benefits and barriers of LA ART, and health systems readiness for LA ART implementation. Because questions regarding LA ART centered on anticipated, hypothetical use given that the product was not yest available in Kenya, we considered the FGD format advantageous over individual in-depth interviews, as it allowed participants to bounce ideas off one another and collectively reason through unfamiliar scenarios. Complete details of FGD procedures, participant consent, and compensation are described elsewhere [24,25].

### Data analysis

We coded transcripts in Dedoose using a combined inductive and deductive approach, and we utilized thematic analysis [27]. Four research assistants (DM, SB, WB, and MM) performed initial coding under the direction of one study team member (SAH) and primary investigators (RP, EW, EA, and CB) through weekly meetings and iterative feedback. SAH prepared an initial codebook based on the FGD guides and her assessment of the first few transcripts. Coding proceeded in three structured rounds to promote consistency across coders. In the first round, all four coders collectively coded one transcript simultaneously, with discrepancies resolved by consensus. In the second round, two coders independently coded a single transcript before meeting as a group to reconcile differences. In the third round, the remaining transcripts were divided for primary coding among the four coders, with SAH reviewing and double-coding all transcripts. Disagreements at each stage were resolved through discussion and consensus, with SAH serving as the final arbiter during independent coding rounds. Following coding, the team conducted an intensive two-day in-person analysis session, in Kenya, using thematic analysis to group codes into themes organized by the SEM framework (individual, interpersonal, institutional health systems, and policy levels). SAH and three Kenyan research assistants jointly conducted this session, developing an analytic codebook with overarching domains, subsequent themes, convergent and divergent subthemes, and illustrative quotes. Themes were finalized collaboratively across coders during this session. The analytic codebook was then used to develop analysis memos guiding separate manuscript efforts. Member-checking sessions were held with all FGD groups between July and November 2022 to validate findings.

Formal intercoder reliability metrics were not calculated; instead, consistency was ensured through the structured multi-round coding process, weekly team meetings, iterative codebook refinement, and double-coding of all transcripts by SAH. Thematic saturation was assessed iteratively throughout coding and confirmed during the two-day analysis session, at which point the team determined that no substantively new themes were emerging across the data. Additional methodological details, including the full coding and analytic process, are available in prior publications [24,25].

## Results

### General characteristics and summary of findings

We held seven focus group discussions with 58 participants representing four stakeholder categories. Healthcare providers took part in two groups (FGD 1, n = 11; FGD 2, n = 8), adolescents and youth living with HIV in two groups (FGD 3, n = 7; FGD 4, n = 7), advocates in two groups (FGD 5, n = 9; FGD 7, n = 7), and policymakers in one group (FGD 6, n = 9). The adolescent and youth participants were evenly split by gender, with half under age 18, and overall ages ranging from 15-24. This group had extensive treatment experience (11 + years on ART), was predominantly enrolled in secondary education, and none reported being married. The healthcare provider group (n = 19, 12 female) brought diverse clinical expertise, including peer mentors, physicians, nurses, nutritionists, and social workers, with most having more than five years of HIV clinic experience and direct involvement in antiretroviral therapy or family planning service delivery. Advocates (n = 16, 10 female) spanned multiple focus areas: HIV prevention, family planning, youth-focused services, key population support, and mental health. The policymaker group (n = 9, 6 female) consisted primarily of individuals working in the public HIV sector. Although stakeholder groups varied in age and professional experience, their FGD responses showed considerable alignment. Where perspectives diverged, we highlight these differences in our findings.

Participant characteristics are summarized in Table 1 [24,25].

**Table 1:** Participant characteristics for the focus group discussions in the KuwaFree! LiveFree! study, Kenya, November 2021–April 2022 (7 focus groups discussions, n = 58 participants).

Variable	Category	N (%)			
Gender		**Adolescent and youth (n = 14)**	**Healthcare Providers (n = 19)**	**Health advocates (n = 16)**	**Policy makers (n = 9)**
	Female	7(50)	12(63.2)	10(62.5)	6(67.0)
	Male	7(50)	7(36.8)	6(37.5)	3(33.0)
Age in year		Below 18 7(50)	25 and below 5(26.4)	Below 25 7(43.9)	Below 40 3(33.3)
		18- 21 6(42.9)	26-35 9(47.5)	25 -30 6(37.7)	40 and above 2(22.2)
		Above 21 1(7.1)	Above 35 5(26.5)	Above 30 3(18.9)	Missing 4(44.5)
Highest level of education	Secondary	11(78.6)	–	–	–
	Some college or higher education	3(21.4) 0	–	–	–
Marital status	Single	14(100)	–	–	–
Occupation		Not employed 11(78.6%)	Clinical Officer 6(31.6)	–	–
		Peer educator 1(7.1)	Nurse -1(5.3)	–	–
		Student 2(14.3)	Nutritionist 1(5.3)	–	–
			Peer mentor- 8(42.1)	–	–
			Research assistant 2(10.5)	–	–
			Social worker 1(5.3)	–	–
Services accessed/offered	HIV treatment	Type of services accessed at the clinic	Type of services provided at the clinic	Thematic focus of advocacy activities	Area of service
		14(100)	14(73.7)	3(18.8)	2(22.2)
	HIV prevention	0	13(68.4)	11(68.8)	2(22.2)
	FP services	0	10(52.6)	5(31.3)	0
	Adolescent and youth	–	–	12(74.1)	1(11.1)
	Other services	0	12(63.2)*	5(31.4)**	2(22.2)
Duration Since ART initiation (years)	=< 10	2 (14.2)	–	–	–
	11-20	6(42.6)	–	–	–
	21+	1(7.1)	–	–	–
	Unknown	4(28.6	–	–	–
Length of time worked			Length of time in the position	Duration worked in the organization	
		–	< 1 year -3(15.9)	< 2 years-1(12.5)	–
		–	1-5 years- 6(31.7)	2-3 years-3(37.5)	–
		–	> 5 years- 10(52.9)	>3 years-4(50)	–
Role in organization	Program implementer	–	–	5 (18.8)	3(33.3)
	Service provider	–	–	4(25)	
	CBO	–	–	9(56.3)	
Sector engaged	NGO	–	–	10(62.5)	
	None	–	–	2(12.5)	
	Private	–	–	3(18.8)	
	Public	–	–	1(6.3)	5(55.5)

Abbreviations: FP = family planning, ART = Antiretroviral Therapy, CBO = Community-Based Organization, NGO = Non-governmental organization.

*Adherence counseling, monitoring viral load, helping in financial support, pediatrics research, retention, training on stigma and disclosure and HIV care, treatment of comorbidities, nutrition education and counseling.

**Key populations, mental health promotion.

Four main themes emerged from the qualitative data analysis, organized across the SEM. At the policy level, parental consent requirements created implementation challenges despite supportive legal frameworks. At the health systems level, themes included provider stigma, inadequate youth-friendly service integration, supply chain gaps, and the need for provider workforce training. At the interpersonal level, themes encompassed peer mentor support and misinformation, parental barriers and education, and variable male partner involvement. At the individual level, adolescent FP access was shaped by myths and misconceptions, fear of procedures and side effects, and preferences for discreet, low-maintenance methods. As LA ART was not yet available in Kenya at the time of data collection, participants discussions of LA ART were anticipatory.

### Policy level: Navigating parental consent requirements in adolescent family planning access

Accessing FP services can present significant challenges for adolescent minors. Despite laws allowing for sexual and reproductive healthcare without parental consent, healthcare providers perceptions or personal beliefs about the need for parental involvement are common barriers to care for minors. Young people often seek services accompanied by peers rather than family members and healthcare providers reported feeling conflicted when offering FP to adolescents without parental involvement. Providers report being concerned about potential backlash from parents who object to FP services being provided to their children without their knowledge, leading to concerns about conflicts between professional responsibility and parental authority. Adolescents and advocates expressed mixed perspectives, in identifying the importance of both independent consent and parental involvement. Balancing the rights of adolescents with parental expectations and influence remains a sensitive issue, complicating FP access for younger individuals.

*“As a health provider you are torn between offering family planning to these adolescents looking at the age and most of the time they come with their peers, they don’t come with their parents. And then at times you find that when a parent discovers that you are giving family planning to their child without their consent, it also becomes a problem.”* **Healthcare Provider, FGD 2**

*“For me I think that is a two-way thing. There must be consent from the adolescents and at some point, since they are still under their parents’ care, there should be consent from their parents.”* **Advocate, FGD 7**

### Health systems level: Creating youth-friendly integrated family planning and HIV services

*Integrating youth friendly clinics with family planning and LA ART services.* Healthcare providers and peer mentors strongly advocated for the integration of FP and HIV services, specifically provision of LA ART, into single, youth-friendly facilities. Participants anticipated that by creating safe, non-judgmental spaces tailored to adolescents unique needs, these integrated services empower young people to independently access contraceptives and HIV care without stigma or barriers. As one youth participant emphasized, these “friendly spaces” are essential because they support young people’s privacy and autonomy. Additionally, a policymaker underlined the importance of ensuring that these services are not only accessible and age-appropriate but also welcoming to adolescents, particularly in the context of LA ART. Integrating FP and LA ART services in a way that resonates with adolescents needs and concerns may help improve the acceptability and uptake of both HIV treatment and FP methods, meeting the needs of young people while enhancing health outcomes for this vulnerable group. By offering comprehensive and integrated services, healthcare facilities can address both HIV treatment and reproductive health in a holistic, adolescent-friendly manner.

*“First there should be friendly spaces for young people to uptake [FP] because you know a lot of young people don’t really go and take FP because of that fear of being judged. But if we have those friendly spaces where adolescents can walk in without a fear of judgment and access this service, I am feeling that is the first step of giving these girls the contraceptive.”* **Adolescent Youth, FGD 3**

“*Where would be the best place to locate the services that is easily accessible to the young people at their convenience so that they don’t feel you are taking them into a clinic that they have felt is not appropriate for their age group? So, the level of integration that can happen within our traditional MCH family planning clinic vis a vis a youth friendly site needs to be taken into consideration so that the acceptability among the targeted population is better.”* **Policymaker, FGD 6**

*Health Systems Readiness for Integrating FP into LA ART Services.* Participants highlighted the importance of system-level support to effectively integrate FP into LA ART services. This integration requires technical support at multiple levels, such as a supply chain to ensure that FP methods are consistently available at the same facility where LA ART is provided, logistical support like storage needs, and private space for provision of all methods. Stock-outs were identified as a significant barrier to effective service delivery of essential contraceptives, particularly short-acting methods like injectable contraception. The integration of FP services into LA ART settings is vital not only to increase access but also to reduce logistical hurdles that often prevent individuals from using FP methods consistently. Participants suggested that a coordinated systems approach could spare individuals from navigating multiple facilities or face interruptions in FP services, which could ultimately improve adherence to both HIV treatment and FP use. Participants cautioned that, without addressing these supply chain issues, integrating FP into LA ART services would be ineffective, limiting the impact of such integration on broader health outcomes.

*“Yes, stock outs have been there. Sometimes we face stock outs especially for the short acting like the Depo.”* **Healthcare Provider, FGD 2**

*“From my experience because we are currently working with the health care workers very closely monitoring family planning health commodities and one of the things that we are experiencing from them is even with the stock availability of the various family planning methods.”* **Advocate, FGD 5**

*Provider stigma and the need for youth-friendly provider training.* Participants described how judgmental attitudes from healthcare providers created an environment of stigma that discouraged adolescents from seeking FP services. Adolescents and youth advocates recounted experiences of being shamed, dismissed, or turned away by providers, reinforcing feelings of embarrassment and alienation. Some providers not only discouraged FP use but also perpetuated myths about contraceptives, which adolescents internalized, leading to confusion, mistrust, and reluctance to return for services.

This provider stigma was so pervasive that adolescents expressed a strong preference for the pharmacy model of FP service delivery, where interactions are straightforward and non-judgmental. As one advocate noted, at pharmacies “*nobody asks you questions*,” in contrast to clinical settings where step-by-step guidance can feel intrusive or judgmental. However, this preference reveals a troubling constraint: pharmacies cannot administer most effective contraceptive methods such as injectable DMPA, implants, or intrauterine contraceptive devices (IUCDs), which require clinical provision.

Participants emphasized that addressing provider stigma requires institutional investment in training. Healthcare providers expressed the need for sensitization on adolescent-friendly services to ensure they can offer non-judgmental, supportive care. This includes understanding the unique needs of adolescents and approaching them in a sensitive, confidential, and unbiased manner. Providers identified this training as a crucial health systems intervention, not merely a personal competency, requiring organizational commitment and resources.

*“We will target all of them, but first of all the information has to be with the health care provider so that they can be non-biased when giving this information.”* **Healthcare Provider, FGD 1**

*“But when you go to a pharmacy, nobody asks you questions, nobody gives you directions, and nobody tells you what to do. You just give them the money, they give you the treatment, you go on with your life. So whatever you do after that is basically you are on your own. But when you go to a hospital and you are with a healthcare worker, they take you step by step and I think most young people because of one, the impatience and also the prejudice to the health care workers they are like ‘I can’t go to hospital, I don’t want to be asked questions, I don’t want to explain myself.”* **Advocate, FGD 5**

*“You know from history, we as adolescents are judged. Someone comes and you are like; ‘Even you? You are planning?’ I mean just how you get handled in the clinic…”* **Adolescent Youth, FGD 3**

*“It depends with the healthcare provider who you get at the facility. Some of them have chased away young girls like you are not supposed to be using these things and most of them are the ones who come up with the myths and they fill them to the kid’s minds. So, it becomes a hard thing because they come and tell you; ‘I was told by the doctor like this’ and so you are like; ‘Which doctor was that one?’ So at times it becomes a bit of conflict because most of them are not youth friendly.”* **Advocate FGD 7**

### Interpersonal level: Family and partner dynamics as barriers and facilitators to adolescent family planning

*The role of peer mentors in FP uptake and awareness among adolescents.* Peer mentors play a crucial role in encouraging FP uptake among adolescents by fostering trust, providing relatable guidance, and sensitizing them to the benefits of contraception. Adolescents are often more receptive to advice from their peers than from older individuals, making peer-to-peer education an effective approach to increasing FP awareness and acceptance. Peer mentors can normalize FP use by sharing personal knowledge and experiences in a non-judgmental way, making FP methods more accessible and appealing. This accessibility fosters an environment where young people can freely ask questions, exchange insights, and explore FP options with confidence. Both adolescents and advocates highlighted the important role of peer mentors in FP education and support.

*“Yes, peer education because you find that I have certain knowledge, my friend here has certain knowledge. Maybe [another adolescent may say] ‘oh, so this is a good method of family planning, maybe I should try’. That is because of peer education. Peer to peer are free with each other.*” **Adolescent Youth, FGD 4**

“*Sensitization, because as we sit like this, we know adolescents are sexually active. So, with or without condoms, they will still engage in sex. But if we do sensitization to improve uptake of FP and give out the effects and side effects of contraceptives, I think there will be a positive impact and it will increase uptake.”* **Adolescent Youth, FGD 4**

*Peer influence can lead to misinformation about FP.* While peer influence is valuable in encouraging FP uptake among adolescents, it also poses risks when misinformation spreads within peer groups. Adolescents may make FP decisions based on their friends’ personal experiences, which can lead to misunderstandings about the effectiveness or side effects of certain methods. Advocates underscored the need for accurate, accessible FP education alongside peer-led support.

*“When it comes to decision-making because you find with peers, they kind of give each other opinions on what is working. So, one person might say I took this, it did not work for me and so others might think that whatever the first one took didn’t work, then it is a bad choice of contraception yet none of the others has tried. So, the peer pressure for lack of a better word, the peer influence might be the downside to it but I don’t think there might be a problem when it comes to uptake. The problem might come when it comes to the peer to peer influence.”* **Advocate, FGD 5**

#### Need for training and accurate information for peer mentors.

Adolescents identified a gap in peer mentor training, highlighting the importance of ensuring peer mentors have accurate, evidence-based information about FP methods. Equipped with this knowledge, peer mentors would be better able to address concerns and provide relevant, credible advice, creating a more supportive environment for peers to make informed decisions about FP.

”*I think [peer education] is a good idea because the peer mentors know their adolescents more than the doctors because they interact more.*” **Adolescent Youth, FGD 3**

*“The peer mentors themselves should have enough information so that they can be able to answer those fears.”* **Adolescent Youth, FGD 3**

*Parents as barriers to adolescents accessing and using FP*. Parents often act as significant barriers to adolescents accessing and using FP due to stigma, misinformation, and a reluctance to acknowledge or seemingly support adolescent sexual activity. Some parents, influenced by personal experience or fears about hormonal effects, actively discourage contraceptive use. The broader reluctance to acknowledge adolescent sexual activity prevents open discussion and leaves adolescents without the guidance needed to access reproductive health services.

*“It is difficult because most of the parents don’t want to see their children sexually active. So they don’t support them in terms of FP. So even those ones who we give [FP to], they really have a lot of stigma especially when they are at home. They hide, they don’t want it to be seen or heard to be on a family planning method. So there is no support.”*

**Healthcare provider, FGD 1**

*“They are few but parents don’t quite often really want to put that idea in their heads that my adolescent child is having sex. The parents have not accepted that the adolescents are having sex. They don’t even want to think that my daughter might be having sex. So you find that is one of the barriers why parents are like why are you taking FP and you are not sexually active?”* **Healthcare provider, FGD 1**

*“I think there is misinformation or maybe fears among some caregivers and so they will not really recommend family planning methods to their children. Some may talk from experience, you know this is an issue to do with hormones. So maybe they experienced some challenges with use of contraceptives and so they do not recommend to their children. “* **Healthcare provider, FGD 2**

*Educating parents on the benefits of FP to gain their support.* While parents can be barriers to adolescent FP use, they also have the potential to be facilitators if they are properly educated on the benefits. Healthcare providers emphasize the importance of engaging parents in conversations about FP to address misconceptions and reduce stigma. Providing accurate information can help shift parental attitudes, making them more supportive of adolescents’ reproductive health needs. One suggested approach is leveraging community health volunteers (CHVs) to facilitate dialogue sessions with parents. These discussions could create opportunities for parents to better understand FP, its role in adolescent health, and the importance of supporting their children’s access to contraception. By using existing community networks, efforts to educate parents can be more effective in fostering acceptance and support for adolescent FP use.

*“I get it, so I think we might as well be having, say dialogues, with the parents, they get to mobilize through CHVs for parents who have adolescents who are sexually active and are eligible to partake in family planning, It is equally important to have dialogue sessions with the parents to just make them understand family planning isn’t bad.”*

**Advocate, FGD 7**

*Male partner involvement in FP decision-making.* Male partners play multifaceted roles in adolescent FP decision-making, ranging from disengaged or unsupportive to actively involved and encouraging. A persistent pattern emerged in which male partners left FP responsibility entirely to female partners, reinforcing gendered power imbalances and limiting shared decision-making. Social pressures around masculinity and fertility could lead to reproductive coercion, with some men actively discouraging long-acting methods. Men’s generally lower health-seeking behavior further contributed to their absence from FP counseling spaces. As a result, women may initiate and use contraceptives without their partners knowledge or support, occasionally leading to disagreements or mistrust.

Despite these challenges, some participants described examples of positive male involvement in FP, where partners take an active role in decision-making and support their girlfriends in accessing contraception. In such cases, men and women approach FP as a shared responsibility, contributing to more informed and mutually respectful choices. This support sometimes included accompanying partners to clinics, inquiring about available methods, and participating in counseling sessions. However, male involvement was not always aligned with equity. Some men exercised dominant influence over FP choices, pressuring their partners into using methods that suited their own desires (e.g., avoiding condoms), regardless of the woman’s preference or comfort. This power imbalance can compromise women’s autonomy and increase their vulnerability to unintended pregnancy or STIs. Taken together, these perspectives reveal the dual nature of male involvement in adolescent FP, both as a barrier and a potential facilitator. While some men perpetuate disengagement or reproductive control, others demonstrate the possibility for supportive, joint decision-making. These insights underscore the importance of engaging male adolescents in FP education and services to foster more equitable partnerships and improve reproductive health outcomes.

*“Actually, it is true. Most of the times the partner, oftentimes the guy, is usually less involved in family planning matters of their partners. You see us boys, or rather men, we usually tend to assume that it is a woman’s responsibility to ensure that they are protected, to ensure that they are safe. So, I will meet a woman and I will expect that she is the one with the responsibility for ensuring that she doesn’t get pregnant.”* **Advocate, FGD 7**

*“For me personally, I can’t advise my girlfriend maybe to use those before we have any other child. We don’t have a child. So for me, the only method I will prefer is using a condom. But maybe we don’t know, maybe your girlfriend is suspecting that you may get her pregnant and maybe she is not ready. So she may look for another family planning method. So you may agree, like she said, for three months.”* **Adolescent Youth, FGD 4**

*“In this study I think reaching out to sexual partners is a very big thing because this responsibility has been left to a lady alone because at some point, we men have poor medical seeking behavior and some, not all, they may not have that mentality of supporting family planning. You can find out that there are some sexual partners that come into disagreements because of family planning. Maybe the boy needs a kid…”* **Advocate, FGD 7**

*“I think for the partners it is almost like a 50/50 thing because there are some who have brought their partners and even they ask for information, at least the best method that their girlfriends can choose or where in the facility they can get family planning, so that they can take their girlfriends. Still again there are those who have brought their partners and them come together. So it is almost like a 50/50 thing.”* **Healthcare provider, FGD 1**

*“So, I think the decision is mostly made by the men especially when it comes to family planning if not the peer because they have an upper hand to play or role to play in terms of decision making.”* **Advocate, FGD 5**

### Individual level: Method-specific fears, myths, and preferences shaping contraceptive choices

*Myths and misconceptions around FP methods that discourage uptake.* Myths and misconceptions surrounding FP and cultural beliefs within society can significantly hinder individuals, especially adolescents, from accessing FP services. Healthcare providers pointed out that while misconceptions among adults can be addressed more easily, adolescents are more susceptible to myths. For instance, there is a prevalent myth in the community that using FP methods, particularly IUCDs, will lead to infertility, cancer, infections, and other problems. Furthermore, cultural norms around sexuality in many African communities create significant barriers for adolescents seeking FP. For example, the use of potentially visible contraception, like the implant, is heavily stigmatized, as it is often seen as an indication that a young girl is sexually active, which can be taboo and lead to judgment and social exclusion. Healthcare providers and advocates recognize that while adolescents may want to protect themselves from unintended pregnancies, they are often constrained by these cultural expectations, where discussing sex and using FP methods is seen as inappropriate for their age and marital status. This cultural stigma complicates the uptake of FP and creates a challenging environment for young people to access the services they need.

“*The adolescents are saying family planning is for people who are already married so that they can plan on their children....*” **Healthcare Provider, FGD 1**

*“Most of them are married and the beliefs and the misconceptions for the adults is a bit easier to deal with them. But for the adolescents, you will find that most of them have the misconception … about the family planning methods like the community are saying when you use family planning you will not have children in future.”* **Healthcare Provider, FGD 2**

*“I am saying injectable are more preferred because it is a method that young girls really relate to. They know how it works since they were young. But if you talk of IUCDs, the thought of them having something inside their reproductive system makes them shun the method. Secondly, they have had a lot of myths concerning IUCDs. One being that it causes rust and it can be infectious and lead to cancers because of the copper and it discourages them from using the IUCD.”* **Advocate, FGD 5**

*“As much as they want to protect themselves, there is this cultural system in them that you should not be seen using family planning and contraceptive if you are not married, so having a young girl with an implant is very difficult because it is one method that one can know you are having especially for women who know how to look,”* **Advocate FGD 5**

*“So sex is more of a taboo in an African setting, not a lot of people talk a lot about sex and this is a young person, let us say 16-17 [years old] picking up condoms. So you will get… they will be judged by the community that you are too young to be having sex. You need to go to school, you need to study, and you need to pass your studies”*

**Healthcare Provider, FGD 1**

*Fear of FP procedures and side effects.* Healthcare providers noted that many adolescents experience significant fear and anxiety about the potential pain and invasiveness associated with certain FP methods, particularly the insertion of IUCDs. In addition to concerns about pain, adolescents often express worry about the side effects of hormonal contraceptives, such as weight gain. These fears can lead to hesitancy in choosing a method and, in many cases, result in adolescents defaulting to the less effective option of condoms. For some adolescents, the fear of weight gain is especially prevalent, with many prioritizing body image concerns over contraceptive effectiveness. This fear of side effects and procedural discomfort underscores the importance of providing comprehensive counseling that addresses these concerns and helps adolescents make informed, confident choices about their contraceptive options.

“*And then others might be very cautious of body shapes like she has talked about the side effects of family planning. They will tell you; ‘I want you to give me something that will not make me fat’ [Laughs] you know because they really still want to maintain that shape because when they look at women who have given birth and maybe they are on a form of family planning and they blame it on the method and they have gained weight, they wouldn’t want to look like that.*”

**Healthcare Provider, FGD 2**

*“But when you look at IUCD which is very intrusive, again they might change their mind because after being explained what the procedure will be undertaken, they opt out of the IUCD because of the procedure of it being intrusive”* **Healthcare Provider, FGD 1**

*Preference for discreet and low-maintenance FP methods among adolescents.* Participants highlighted the unique challenges adolescents face in selecting FP methods, often navigating privacy concerns, ease of use, and accessibility. A recurring theme among healthcare providers was the importance of discretion for adolescents, many of whom seek to prevent partners or family members from discovering their use of contraception. Adolescents often favored methods that were not only effective but also less visible and more convenient to manage. Healthcare providers noted that daily pill-taking posed adherence challenges, especially for adolescents living with HIV who experience “pill fatigue,” or who live in shared households with limited privacy. Injections and implants were more commonly preferred due to their discreet nature and longer duration of action, reducing the need for frequent clinic visits and minimizing the risk of detection. Visibility of the method also emerged as a key concern, particularly for adolescents who were hesitant about methods that might leave marks or be associated with pain. Even with proper counseling, some adolescents declined injections due to discomfort or the burden of repeated clinic visits. Additionally, adherence to clinic return dates posed another barrier. Adolescents using methods like injectable contraception often prioritized discretion and financial constraints over strict adherence to recommended schedules, which could result in inconsistent use and unintended pregnancy.

*“I feel young people might be open to, and with the ones that I have interacted with, more want to go with a more discreet method of family planning in that they are open to implants, they are open to injections but when it comes to daily pills, a lot of them have the pill fatigue in that sometimes they might forget, sometimes they are afraid if they are living in a household that is shared. So most of them don’t like pills. They prefer a form of family planning that is discreet, nobody knows they are on it and it is easier for them. So the injection I think works better for most of them.”* **Advocate, FGD 5**

*“They have actually raised concerns about the visibility and the one that is not visible. But after counseling and they look at the frequency of coming to the clinic and others don’t want to feel pain like every time they come they would not prefer that injection.”* **Healthcare Provider, FGD 2**

*“Another thing is adherence to the scheduling of that family planning. If it is Depo, they will only come at their convenient time and not necessarily as per the return to clinic date because if it is done in secret, then it means they will only come when they have the money and they are in a position to come back for a revisit…”* **Healthcare Provider, FGD 2**

## Discussion

This qualitative study examined barriers and facilitators to family planning access among adolescents in Kenya through the perspectives of four key stakeholder groups: adolescents and youth living with HIV, healthcare providers, advocates, and policymakers. Our findings reveal that contraceptive access for AGYWLHIV is shaped by multi-level factors spanning individual preferences, interpersonal relationships, health system characteristics, and policy environments. Critically, these findings are situated within the anticipated introduction of LA ART in Kenya, which creates new and distinct opportunities to integrate FP services within HIV care delivery in ways that oral ART has not previously enabled. At the individual level, AGYWLHIV expressed strong preferences for discrete, low-maintenance contraceptive methods that minimize visibility and reduce the burden of daily adherence, preferences that converge directly with the injection-based, low-frequency delivery model of LA ART. Interpersonally, relationships demonstrated dual roles: while peer mentors and some partners facilitated contraceptive decision-making, parental attitudes and partner knowledge gaps often created barriers. These interpersonal dynamics will be central to whether LA ART-FP co-delivery is accepted and sustained by adolescents and their social networks. Within health systems, provider bias, inadequate youth-friendly infrastructure, and fragmented service delivery emerged as persistent obstacles, all of which must be addressed to realize the potential of LA ART clinic visits as integrated FP delivery platforms. At the policy level, despite Kenya’s supportive legal framework allowing adolescent access to reproductive health services without parental consent, implementation gaps driven by provider uncertainty and resource constraints limit actual access. Addressing these gaps is essential groundwork for any LA ART-FP integration strategy to succeed at scale. These findings underscore how AGYWLHIV navigate intersecting vulnerabilities related to both HIV management and pregnancy prevention, facing compounded barriers that demand integrated, multi-level solutions.

1. Individual Level

At the individual level, our findings reveal that adolescents’ contraceptive choices are significantly influenced by concerns about privacy, convenience, and social stigma. Participants consistently expressed preferences for discreet, low-maintenance FP methods, particularly injectables and implants, that could be used without detection by parents or sexual partners. These preferences are directly relevant to LA ART integration; the injection-based delivery of LA ART mirrors the modality of injectable contraception, and the low-frequency dosing schedule of LA ART addresses the pill fatigue and daily adherence burden that currently drive adolescents away from oral contraceptive methods. The anticipated introduction of LA ART therefore represents a convergence point, an opportunity to normalize injection-based, long-acting methods for both HIV treatment and FP simultaneously, within the same clinical encounter.

These findings align with extensive prior research demonstrating that method preference among adolescents is primarily shaped by considerations of convenience, side effects, and confidentiality rather than solely by contraceptive effectiveness [28,29]. Research similarly found that some adolescents preferred covert methods, such as injectables, specifically due to their ability to hide contraceptive use from parents and sexual partners [30]. The desire for privacy and confidentiality stems from the persistent stigma surrounding contraceptive use and premarital sex among unmarried adolescents, creating barriers that extend beyond simple access to methods [31,32]. Furthermore, our participants concerns about pill fatigue, fear of judgment, and inability to afford frequent clinic visits, echo findings that self-injectable contraception and other low-maintenance methods may offer solutions to the particular access challenges faced by unmarried adolescents [30,33]. These challenges include not only privacy concerns but also practical barriers such as the cost of travel, distance to health facilities, financial dependence on parents, and inconvenient clinic hours that conflict with school attendance [34,35]. Additionally, the fear of procedural pain, particularly regarding IUCD insertion, represents another significant individual-level barrier that influences method selection. Recent U.S. Centers for Disease Control and Prevention recommendations emphasize pain control options for IUCD placement, including lidocaine administration through paracervical blocks or topical application, representing a relatively small but significant shift toward patient-centered care [36]. These findings underscore the critical need for tailored FP counseling and education specifically designed for adolescents, delivered within youth-friendly clinical settings that prioritize privacy, comfort, and non-judgmental care. Long-acting reversible contraceptives coupled with LA ART represent a particularly promising option for this population; by offering both HIV treatment and FP through a shared injection-based modality, LA ART delivery platforms can address the practical and emotional concerns that currently drive adolescents toward or away from specific contraceptive methods, while reducing the compounded burden of managing both conditions separately [37,38].

2. Interpersonal Level (Parents, Partners, Peers)

At the interpersonal level, our findings revealed that relationships play a complex dual role in adolescent contraceptive decision-making, with some relationships empowering autonomous decisions while others constrained reproductive agency. Three key relationship types emerged as critical influences: parental relationships, sexual partnerships, and peer networks.

Parental involvement presents complex dynamics in contraceptive access. Research from the United States shows mandated parental involvement laws create significant obstacles, with only slightly more than half of adolescents willing to obtain contraceptives if parental notification were required [39]. In Kenya, qualitative research reveals similar parental barriers, with adults often viewing contraception as inappropriate for unmarried adolescents, creating stigma and limiting open communication about sexual and reproductive health [40]. However, younger adolescents are more likely to have parental awareness of clinic visits, suggesting parental involvement can be beneficial when voluntary and supportive [39]. These findings highlight the need for parental sensitization campaigns to address contraceptive misconceptions and normalize family discussions about sexual health. Furthermore, in the context of LA ART, parental attitudes will be particularly consequential for younger AGYWLHIV who remain under parental care, as parental objection or unawareness of regular injection visits could directly undermine adherence to LA ART-FP co-delivery services.

Male partner involvement emerged as multifaceted, simultaneously serving as both facilitator and barrier to contraceptive use. Partners’ involvement ranged from complete disengagement to active participation in contraceptive decision-making. These dynamics are consistent with Connell’s Theory of Gender and Power, as applied by Wingood and DiClemente to women’s HIV and reproductive health, which identifies gendered power imbalances, including the delegation of contraceptive responsibility to women and male dominance in sexual decision-making, as structural determinants of women’s reproductive vulnerability [41]. Women in committed relationships reported their male partners as active participants in the decision process, providing information and contributing to discussions on family size and timing [42–44]. Well-counseled and educated male partners can increase contraceptive use, and relationship context significantly influences contraceptive uptake, with adolescents in committed relationships demonstrating higher contraceptive use compared to casual encounters [39,44]. However, male partners can also create substantial barriers. Many lack knowledge of contraceptive methods, especially long-acting reversible contraception (LARC), promoting misinformation and perpetuating misconceptions that deter use of these methods [44]. These findings underscore the need for interventions that engage male partners in comprehensive contraceptive education, particularly regarding LARC methods, to foster equitable partnerships, reduce misinformation, and improve reproductive health outcomes. For AGYWLHIV specifically, these relational power dynamics are further compounded by HIV status. Fear of inadvertent disclosure to a partner, stigma within relationships where HIV status is known or suspected, and the added negotiating burden of managing both contraceptive use and ART adherence without partner support can significantly erode reproductive autonomy. AGYWLHIV may be less able to assert contraceptive preferences or attend clinic visits independently when partner dynamics are characterized by control or mistrust, underscoring the need for interventions that address HIV-related relational vulnerabilities alongside general contraceptive education [41,45]. Furthermore, as LA ART programs are rolled out, male partners’ understanding and attitude towards injection-based treatment may similarly influence whether AGYWLHIV can consistently attend or receive LA ART-FP co-delivery services without conflict.

In contrast to the variable influence of parents and partners, peer mentors and youth advocates consistently emerged as facilitating actors, serving as trusted sources of contraceptive information and support. This positive influence reflects broader evidence supporting peer influence in adolescent health behaviors [46,47]. Systematic reviews show peer education interventions demonstrate positive changes in knowledge, attitudes, and self-efficacy related to contraceptive use and HIV prevention, though behavior change results are mixed [46,47]. Effectiveness depends on implementation factors and specific peer educator roles [47]. These findings support expanding peer mentor-led outreach programs with evidence-based training that incorporates relational dynamics beyond information-only approaches. Furthermore, peer mentors who are familiar with LA ART could also play a particularly powerful role in normalizing LA ART and injectable FP among their fellow peers. They may serve as early adopters to this service and further advocates who can counteract misinformation while building confidence in integrated LA ART-FP services.

Collectively, these findings suggest three critical programming implications: implementing parental sensitization campaigns to address contraceptive misconceptions and normalize family discussions about sexual health, engaging male partners in contraceptive education to foster equitable partnerships and improve reproductive health outcomes, and expanding peer mentor-led outreach programs with evidence-based training. Critically, these interventions should be designed as gender-transformative rather than merely gender-sensitive, moving beyond information provision toward approaches that actively challenge the gendered norms, power imbalances, and social expectations that underlie the relational barriers identified in our study. This includes engaging male adolescents in structured dialogues around shared contraceptive responsibility, masculinity, and reproductive decision-making, and embedding these approaches within LA ART-FP integrated platforms where both partners may be present or reachable. Each of these interpersonal dynamics will also shape the uptake of LA ART-FP integrated services: parental attitudes toward clinic visits, partner support or resistance to long-acting injectable methods, and peer influence on adolescents’ willingness to initiate or continue LA ART will all be critical determinants of whether integrated programs succeed for AGYWLHIV.

3. Institutional Level (Health System/Facility)

At the health systems level, our findings highlight two intersecting institutional failures: provider stigma that actively discourages adolescent FP-seeking, and structural gaps in service integration that create logistical barriers even when adolescents are willing to seek care. Participants across stakeholder groups described healthcare providers, particularly nurses, as gatekeepers who shamed, dismissed, or misinformed adolescents, driving young people to prefer pharmacy-based services despite pharmacies’ inability to provide most effective contraceptive methods. Alongside this, supply chain gaps and the absence of private, youth-friendly spaces within facilities further undermined service delivery. These findings point to an institutional environment where the barriers are not merely attitudinal but embedded in how services are organized and resourced. This is consistent with broader evidence showing that youth-friendly services improve contraceptive uptake when providers receive training in adolescent-centered care, trained providers demonstrate significantly lower age bias, and facilities implementing adolescent-responsive approaches report increased uptake among young people [46–48]. Evidence identifies seven key elements of youth-friendly service delivery, nonjudgmental care, confidentiality, comprehensive method availability, affordability, convenient hours, respectful staff, and age-appropriate environments, all of which were identified as gaps by participants in our study [49–51]. Additionally, structural modifications, such as private consultation rooms, confidential intake processes, separate waiting areas, and systems ensuring billing confidentiality, enhance youth-friendliness beyond provider training [49,50,52]. Critically, our findings suggest that in the Kenyan context, provider stigma is the most proximal barrier, one that undermines all other structural improvements if left unaddressed.

Integration of FP with HIV services offers particular advantages for youth by providing a “one-stop shop” model that reduces barriers inherent in fragmented care delivery. Rather than requiring multiple appointments across different clinic locations, increasing transportation costs, time burden, and disclosure risks, integrated service delivery allows for comprehensive reproductive health and HIV services during a single visit. The introduction of LA ART is particularly significant in this regard, unlike oral ART, which is largely self-administered with infrequent clinic contact, LA ART requires regular scheduled injection visits, creating structured and predictable clinical touchpoints that did not previously exist under differentiated service delivery models. These visits represent a natural and recurring opportunity to co-deliver FP services, counsel on long-acting contraceptive methods, and address reproductive health needs, without requiring adolescents to navigate additional appointments or disclose their HIV status in separate clinical settings. In this way, LA ART fundamentally reshapes the service delivery landscape for AGYWLHIV, transforming routine HIV care visits into comprehensive reproductive health encounters.

Overall, these findings indicate that facilities must invest comprehensively in provider training emphasizing adolescent-centered care, establish adolescent-friendly structural changes, and integrate FP with HIV care and other youth services. As LA ART programs are developed and rolled out in Kenya, embedding FP service delivery within these platforms from the outset, rather than adding it later as a parallel system, will be essential to maximizing their impact for AGYWLHIV.

4. Policy Level

Kenya’s legal framework distinguishes several concepts that are often grouped under the age of consent. The age of majority is 18 under Article 260 of the Kenyan Constitution (2010), which defines a child as any person under the age of 18 [53,54]. The age of sexual consent is also 18 under Sexual Offences Act (2006) [53,54]. Although Kenya has comprehensive guidelines and policies on adolescent health care, including the National Reproductive Health Policy 2022–2032, they still require parental consent before minors can receive reproductive health services [53,54].

Our findings reveal significant policy-level barriers affecting adolescent FP access, with parental consent requirements creating complex implementation challenges despite existing supportive legal frameworks. Kenya has developed an extensive adolescent SRH policy architecture, including the Adolescent Reproductive Health and Development Policy (2003), the National Adolescent Sexual and Reproductive Health Policy (2015), and its Implementation Framework (2017–2021), all of which support youth-friendly reproductive health services, sexuality education, and increased funding for adolescent health [54,55]. These policies support adolescent access, but parental consent requirement limits it. As most adolescents seeking FP services are under 18, the consent provisions above determine whether these supportive policies translate to independent access [53,54].

Providers’ uncertainty about whether they may serve minors without parental involvement, and their concern about parental backlash, reflect the formal consent requirement and personal beliefs about adolescent sexuality and contraception. Both dimensions are evident in our data: providers feeling “torn” between supporting adolescents and deferring to parental authority, and concerns about backlash from parents objecting to FP services provided without their knowledge. These implementation barriers are compounded by cost and geographic access challenges, as evidenced by 25% of AGYW having unmet need for FP in Kenya, with rural women being almost 4 times more likely to experience unmet need compared to urban counterparts [56].

Kenya’s situation exemplifies a well-documented pattern in adolescent SRH policy globally: the gap between progressive policy architecture and its implementation is not incidental but structural [57]. Despite the existence of comprehensive national frameworks, county-level development plans have largely failed to allocate adequate funding for adolescent reproductive health, leaving the policy commitments on paper without the resources to make them real in practice [55]. Cost represents the most significant structural barrier, with long-acting reversible contraceptives like IUCDs being most cost-effective at less than 5 USD per couple year protection while upfront cost barriers particularly affect youth accessing these methods [57]. The tension between adolescent autonomy and parental authority reflects broader challenges in translating policy into practice, where healthcare providers navigate conflicting professional responsibilities. Geographic disparities further compound these challenges, as women in high-poverty communities face 2 times higher likelihood of unmet need, highlighting how multiple policy-level barriers intersect to limit adolescent access [57]. These findings underscore how legal provisions alone are insufficient without addressing implementation barriers and resource constraints.

Policymakers should prioritize comprehensive approaches that address both legal implementation and structural barriers simultaneously. Critical policy actions include developing provider guidance that addresses the legal complexity around adolescent consent while establishing minimum standards for youth-friendly FP service delivery, creating frameworks for parental engagement that distinguish supportive involvement, overall ensuring sustainable funding with county-level accountability mechanisms, and implementing removal of cost barriers for economically vulnerable adolescents. Additionally, the anticipated rollout of LA ART in Kenya represents a timely and strategically significant policy opportunity to address the dual burden of unintended pregnancy and HIV management that AGYWLHIV face. Unlike oral ART programs, LA ART’s injection-based delivery model and required visit schedule create a built-in infrastructure for FP co-delivery that policymakers can leverage from the outset. Embedding FP into LA ART policy frameworks now, including provider guidelines, supply chain planning, and facility readiness standards, will ensure that these systems are designed for integration rather than retrofitted later. This approach maximizes resource efficiency, normalizes contraceptive use within routine HIV care, and addresses the shared barriers that currently limit both ART adherence and FP uptake among AGYWLHIV. Multi-year budget commitments are essential to enable long-term planning for comprehensive FP programming that addresses rural-urban disparities and supports provider training on adolescent-friendly service delivery that respects both autonomy, family dynamics, and facilitates integrated service deliver models [56,57].

## Limitations

This study has several limitations that should be considered when interpreting the findings. The policy level analysis was not particularly well-developed, lacking detailed exploration of specific policy mechanisms, implementation frameworks, or comprehensive analysis of how existing policies could be reformed to better serve adolescents. This could be due to our policymaker sampling including largely HIV-related programmatic focus. Related, our sampling excluded parents or guardians, whose perspectives are key, as our own findings underscore their role in FP decision-making for AGYW. In addition, the AYPLHIV FGDs included both male and female participants and were not stratified by sex, which may have limited female participants willingness to share personal contraceptive experiences. Furthermore, as LA ART is not yet available in Kenya, participants perspectives on LA ART and its integration with FP were hypothetical and may not reflect how adolescents, providers, and families response once services are implemented. The study relied on focus groups alone. Individual in-depth interviews may have elicited more candid accounts of personal contraceptive use and HIV-related disclosure concerns, and future studies should combine both methods. Lastly, the study design itself presents limitations that could affect the transferability of the findings to other settings within Kenya or outside. We acknowledge that Rafiki Clinic represents an optimized care environment, with existing FP-HIV integration, dedicated youth-friendly spaces, and trained peer mentors, that is atypical of most public-sector HIV clinics in limited-resource settings. As a result, our findings likely reflect a best-case scenario, and the barriers we identified may be underestimated relative to what adolescents’ experience in routine care settings. Additionally, the supportive, youth-friendly environment of Rafiki Clinic may have influenced participant responses, particularly among AYPLHIV recruited during scheduled clinic visits; adolescents receiving care in an integrated, non-judgmental setting may report more positive experiences with FP access and provider interactions than those attending less supportive facilities, potentially underrepresenting the severity of barriers experienced elsewhere.

With respect to scalability, peer mentor programs and provider training in adolescent-centered care represent the most immediately transferable lessons from this study, requiring relatively modest investment and integration into existing health worker training frameworks and HIV clinic structures. Structural facility modifications, supply chain overhauls, and full FP-HIV service integration require greater institutional investment and are more dependent on sustained domestic funding and political commitment. The anticipated LA ART rollout presents a strategic window for embedding scalable FP integration components, particularly peer mentor engagement and provider sensitization, within new program infrastructure from the outset, rather than retrofitting them later.

## Conclusion

Addressing the multi-level challenges identified in this study requires comprehensive interventions that acknowledge the unique position of AGYWLHIV. The anticipated rollout of LA ART presents a timely opportunity to address these multi-level challenges through integrated service delivery. As LA ART infrastructure is developed to overcome barriers facing AGYWLHIV including stigma, confidentiality concerns, and access challenges, integrating long-acting contraceptive methods into these emerging systems can maximize efficiency and address the dual burden of unintended pregnancy and HIV management. This requires comprehensive interventions that combine policy reform supporting integrated service models, health systems strengthening that leverages LA ART infrastructure investments, community engagement addressing interpersonal barriers, and sustainable financing to improve reproductive health outcomes for Kenya’s adolescent population.

## Supporting information

S1 File Demographic questionnaire. Questionnaire used to collect demographic characteristics of focus group participants.
(DOCX)

S2 File Focus group discussion guide for adolescents and young people living with HIV. Semi-structured guide used in focus group discussions with adolescents and young people living with HIV.
(DOCX)

S3 File Focus group discussion guide for healthcare providers. Semi-structured guide used in focus group discussions with healthcare providers.
(DOCX)

S4 File Focus group discussion guide for advocates. Semi-structured guide used in focus group discussions with advocates.
(DOCX)

S5 File Focus group discussion guide for policymakers. Semi-structured guide used in focus group discussions with policymakers.
(DOCX)
