# Supplementary material for: Addressing barriers and facilitators to family planning uptake among adolescent girls and young women in the anticipated context of long-acting antiretroviral therapy (LA ART)
Source: PLOS Glob Public Health. 2026 Nov 10;6(11):e0007546. doi: 10.1371/journal.pgph.0007546 (PMC13750552; doi:10.1371/journal.pgph.0007546)
Supplement: Supplementary file 1 [file pgph.0007546.s001.docx]

**Co-benefits of co-delivery of long-acting antiretrovirals and contraceptives**

**Demographic Questionnaire**

**Participant’s ID:** XXXXX	**Date completed:** DD/MMM/YY

Study Staff Initials: __________

| Question | Response |
| --- | --- |
| 1. What is your date of birth? | __ __/__ __ /__ __ (DD/MMM/YY) |
| 2. What is your marital status? | a. Married and co-habiting b. Married but not co-habiting c. Not married but co-habiting d. Single e. Widowed f. Divorced g. Separated h. Other (Specify: __________________) |
| 3. What is the highest level of education you completed? | a. None b. Some primary school c. Completed primary d. Some secondary school e. Completed secondary school   d. Some college or higher education |
| 4. Please indicate your language proficiency | a. Kiswahili b. English c. Other (Specify: __________________) |
| 5. Choose your proficiency level in speaking language chosen above 6. Choose your proficiency level in reading language chosen above 7. Choose your proficiency level in writing language chosen above | a. Basic b. Intermediate c. Advanced d. Basic e. Intermediate f. Advanced g. Basic h. Intermediate i. Advanced |
| 8. What is your ethnic background? | a. Luo b. Luhya c. Kikuyu d. Embu e. Kalenjin f. Kamba g. Kisii h. Maasai i. Mijo Kenda j. Other (Specify: __________________) |
| 9. Where is your homestead? | a. Uasin-gishu District b. Elgeyo Marakwet c. Nandi District d. BaringoDistract e. Kericho District f. Bungoma District g. Other (Specify: __________________) |
| **Financial information** | |
| 10. Do you have an occupation? | a. Yes b. No |
| 11. Please specify your occupation ? | a. House wife b. Domestic worker c. Saloonist d. Farmer e. Fish monger f. Hotelier g. Health care worker h. Vegetable vendor i. Sales j. Security k. Small business owner l. Teacher m. Construction n. Other (Specify: _____________________) |
| 12. Are you currently employed? | a. Yes b. No |
| 13. What were your sources of income or other financial or material support in the past month? *(Mark all that apply)* | a. Formal employment b. Self-employment c. Government or social grant d. Family members e. Partner/s f. Friends g. Other (Specify: __________________) |
| 14. What is your monthly household income? *(This will be an estimated total in KSH for everyone in the household)* | a. ___________ |
| 15. Who do you live with? *(Mark all that apply)* | a. Live alone b. With parent(s) c. Spouse or partner d. Children e. Relatives f. Friend(s) g. Other (specify) |
| 16. How many overall (biologic and non-biologic) children do you care for? | a. 0 b. 1 c. 2 d. 3 e. 4 f. 5 g. >5 (Specify:______________) |
| 17. In total, how many people do you live with? (Do not include yourself) | a. None b. 1 c. 2 d. 3 e. 4 f. 5 g. 6 h. 7 i. >7 (Specify:___________) |
| **Contact Information** | |
| 18. Please provide us with your postal address | _____________ |
| 19. What is the closest landmark next to your home? | ____________ |
| 20. Can we reach you via mobile phone? | a. Yes b. No |
| 21. Mobile number   Who does phone belong to? |  |
| 22. Have you shared your HIV status/Family planning details with family? | a. Yes b. No |
| 23. If we are unable to reach you, can we leave a message with whoever answers the phone? | a. Yes b. No |
| 24. Can text message be sent to this phone? | a. Yes b. No |
| 25. Is there another number we can reach you at? | a. Yes b. No |
| 26. Mobile number 27. Who does phone belong to? | a. |
| 28. Have you shared your HIV status/Family planning details with family? | a. Yes b. No |
| 29. If we are unable to reach you, can we leave a message with whoever answers the phone? | a. Yes b. No |
| 30. Can text message be sent to this phone? | a. Yes b. No |
