# Supplementary material for: Addressing barriers and facilitators to family planning uptake among adolescent girls and young women in the anticipated context of long-acting antiretroviral therapy (LA ART)
Source: PLOS Glob Public Health. 2026 Nov 10;6(11):e0007546. doi: 10.1371/journal.pgph.0007546 (PMC13750552; doi:10.1371/journal.pgph.0007546)
Supplement: Supplementary file 2 [file pgph.0007546.s002.docx]

**Focus group discussion guide for adolescents and youth**

“My name is _______________. Thank you all for your willingness to be a part of this discussion. I am part of the Kuwa Free study. The main goal of this discussion is to better understand your feelings and choices regarding HIV treatment medications or antiretroviral therapy (ART) and family planning or contraceptive options to prevent unplanned pregnancy. I am looking forward to hearing your thoughts on HIV treatment (or antiretrovirals), especially long-acting antiretroviral therapy (LA ART), and family planning (or contraceptives). Please know there are no right or wrong answers and we welcome every opinion about the topics we will discuss, so feel free to share your thoughts, opinions and views openly. If during our discussion, there are issues or concerns that you would like to talk about, feel free to bring them up, even if I don’t ask about them. If you have specific questions during the interview, I will take note of them and answer them directly after the interview. If I cannot answer them, I can refer you to someone who may be able to help. Before we start, can you confirm that you have already provided written informed consent to take part in this discussion? [Wait for oral confirmation to begin].

Despite the audio recorder, I want to remind you all that what we discuss here will be kept confidential, and that we will not share your personal information or responses with anyone outside of the study. You will not be judged by your responses. This discussion will take approximately 60 minutes.

Does anyone have any questions before we get started? Do you all agree to have the discussion audio recorded?”

*Interviewer: switches recorder on*

“Okay, let’s begin.”

***(Interviewer: Ensure the interviewees’ demographics are recorded and assign IDs. Provide date, group being interviewed, time, study ID of all participants being interviewed.)***

**Domain 1: Experience with ART, including beliefs and perceptions**

“To start this up, I will ask about your experiences with HIV drugs”

***Drug related barriers to ART use***

What are some of the challenges specific to ART drugs that make them difficult to adhere to?

***Drug related facilitators for ART use***

What factors would make ART drugs easier to adhere to?

**Domain 2: Family Planning**

“Thank you all for such a good discussion so far. I am now going to transition us to the second section that focuses on family planning. For the purposes of this discussion, when we say family planning, we mean both counseling and provision of methods to delay or prevent pregnancy. Common examples of family planning methods in our setting include condoms, oral pills, injectable FP, implants, and IUDs.”

- What family planning methods do adolescents prefer to take up? Why do they prefer those methods? Why do they not prefer the methods not preferred?
- What are the myths and stigma related factors surrounding use of family planning methods amongst adolescent girls and young women?
- Who are involved in the decision making process for adolescents? How do they get involved e.g. by the adolescent or by the health care worker/ school staff etc.? Why are they involved? At what point are they involved?
- What family planning methods are commonly offered to adolescents at clinics?
- What are your thoughts about long term family planning methods that are given as implants?
- What are your thoughts about the ones that are inserted in the uterus?
- What are your concerns about adolescent who are on ARVs while also using family planning?

**Domain 3: Long-acting ART use**

“We are now going to move onto the third section of our discussion. Long-acting antiretrovirals (ARVs), such as injectable ARVs are being investigated in substitution of currently available oral ARVs. These will be every 1-2 monthly injections that will allow users to avoid daily oral antiretroviral therapy. Long-acting ARVs are also under study for prevention, but for our time today we will focus on long-acting ARVs for treatment only.”

- Tell us first what you know or have heard about injectable ARVs?
- Do you believe that this form of HIV treatment will be acceptable to adolescents as an alternative to oral treatment? Tell us more in terms of how comfortable adolescents will be with injectable ART
- Now tell us how complicated or difficult its use will be
- Do you think adolescents in the 15-24 age group who are currently using ART would prefer to use injectable ART rather than oral ART? Why or why not? How might these factors differ between adolescents vs. adults?
- In general, how do you think adolescents who choose to use injectable ART might be different than those who would not want to use injectable ART? *(differences in ART adherence, awareness of ART options, schooling differences e. g primary vs secondary vs colleges and universities, boarding versus day, financial differences, caregiver differences )*
- Do you think health care workers will prefer to give injectable or oral ARVs to adolescents? Clarify
- How about parents? What do you think they will prefer and why/why not?
- What adjustments would clinics have to make to be able to give injectable ART to adolescents?

*Benefits*

- What benefits do you foresee with injectable ART use among adolescents compared to oral ART?
- Do you think adolescents would have better adherence using injectable ART compared with oral ART? Why or why not?
- What adjustments would adolescents and their families have to make in-case of injectable ART?

*Anticipated challenges or drawbacks*

- What challenges would adolescents face with injectable ART?

🡪*Potential probes:*

- *Fear of jabs*
- *Handling side effects, drug-drug interactions*
- *Transportation costs, travel time*
- *School or holiday schedules*
- *Provider training/attitudes*
- *Facility logistics, supply chain, stockouts*
- What challenges would parents face with injectable ART?
- How about the health facilities? What challenges would they face?
- Why would some adolescents refuse injectable ART as a form of treatment?
- Is there anything providers or clinics can do to improve the patients’ experience with injectable ART to make this treatment successful for them?

**Domain 4: Long-acting ART and FAMILY PLANNING**

“For the next section, I want to ask about your thoughts of where there might be benefits or challenges in the use of long-acting ART and long-acting family planning. When we say long-acting family planning, we mean IUDs, implant, and 3 monthly injections”

- What benefits would be there for an adolescent who chooses both the injectable ART and long acting contraception?
- What concerns would an adolescent who is on injectable ART have when they need contraception?
- What concerns would an adolescents who is on a contraception have about using injectable ART?
- Which would be better to the adolescents, to give family planning in an HIV clinic, or to give injectable ART in a family planning clinic? Why?

Thank you very much for your time and responses to the questions.”
