# Supplementary material for: Addressing barriers and facilitators to family planning uptake among adolescent girls and young women in the anticipated context of long-acting antiretroviral therapy (LA ART)
Source: PLOS Glob Public Health. 2026 Nov 10;6(11):e0007546. doi: 10.1371/journal.pgph.0007546 (PMC13750552; doi:10.1371/journal.pgph.0007546)
Supplement: Supplementary file 3 [file pgph.0007546.s003.docx]

**Focus group discussion guide for healthcare providers**

“My name is _______________. Thank you all for your willingness to be a part of this discussion. I am part of the Kuwa Free study. The main goal of this discussion is to better understand your feelings and choices regarding HIV treatment medications or antiretroviral therapy (ART) and family planning or contraceptive options to prevent unplanned pregnancy. I am looking forward to hearing your thoughts on HIV treatment (or antiretrovirals), especially long-acting antiretroviral therapy (LA ART), and family planning (or contraceptives). Please know there are no right or wrong answers and we welcome every opinion about the topics we will discuss, so feel free to share your thoughts, opinions and views openly. If during our discussion, there are issues or concerns that you would like to talk about, feel free to bring them up, even if I don’t ask about them. If you have specific questions during the interview, I will take note of them and answer them directly after the interview. If I cannot answer them, I can refer you to someone who may be able to help. Before we start, can you confirm that you have already provided written informed consent to take part in this discussion? [Wait for oral confirmation to begin].

Despite the audio recorder, I want to remind you all that what we discuss here will be kept confidential, and that we will not share your personal information or responses with anyone outside of the study. You will not be judged by your responses. This discussion will take approximately 60 minutes.

Does anyone have any questions before we get started? Do you all agree to have the discussion audio recorded?”

*Interviewer: switches recorder on*

“Okay, let’s begin.”

***(Interviewer: Ensure the interviewees’ demographics are recorded and assign IDs. Provide date, group being interviewed, time, study ID of all participants being interviewed.)***

**Domain 1: Experience with ART, including beliefs and perceptions**

“To start this up, I will ask about your experiences with HIV care in the first section.”

- What is your role in the care of people living with?
- How long have you been caring for adults living with HIV? What has your experience been like to date?
- How long have you been caring for adolescents living with HIV? And what has your experience been like caring for adolescents?
- How have those experiences caring for adolescents been different than caring for adults? In your opinion, what are some differences between caring for adults vs. adolescents living with HIV? *Potential probes:*
  - Nuances to disclosure, stigma
  - Issues with adherence, including due to residential schools

***Barriers to ART use***

- From your experiences interacting with adolescents, what barriers do they generally face when taking ARVs? Are those any different from adults?

*🡪 Potential probes:* Describe any barriers associated with:

- ***individual-***
- adherence
- forgetting to take ART
- alcohol use, other drug use
- lack of food
- acquired HIV drug resistance
- drug-drug interactions
- ***interpersonal-***
- disclosure
- stigma
- lack of privacy
- family/partner support
- peer support
- provider attitudes
- ***organizational-***
- provider training
- provider strikes
- drug stock outs
- transportation to facilities
- school and holiday schedules
- costs of coming to facilities/missing school/work
- travel time, or long waits at clinics
- ***societal/cultural norms-***
- adolescent autonomy
- cultural norms for adolescents taking lead for their health
- gender norms around ART use
- ***policy/structural-***
- legal status of adolescents
- discrimination against those living with HIV
- unemployment and scarcity of financial resources, poverty

***Facilitators for ART use***

- From your experiences interacting with adolescents, what specific factors facilitate use of ARVs for adolescents? Are those any different from adults?

*🡪 Potential probes: Describe any facilitators associated with (use same as above list):*

**Domain 2: Family Planning**

“Thank you all for such a good discussion so far. I am now going to transition us to the second section that focuses on family planning. For the purposes of this discussion, when we say family planning, we mean both counseling and provision of methods to delay or prevent pregnancy. Common examples of family planning methods in our setting include condoms, oral pills, injectable DMPA, implants, and IUDs. There are many more methods but these are some of them.”

- What type of family planning services does this facility provide? What types of family planning methods are offered at this facility? For example, pills, injection, implants or IUCDs?
- Can you describe your experiences in offering family planning services? Any differences in offering family planning services to adolescents girls vs. young women vs. older women? What about differences between girls or boys, or women or men?
- What problems have you faced in offering family planning and have your patients experienced in getting family planning? Anything specific to adolescents girls or young women? *Potential probes: lack of time to counsel adequately, misinformation in community or from parents, belief that adolescents are not or should not be having sex, conflicting religious beliefs around family planning use, provider bias against offering long-acting family planning methods to adolescents*
- What family planning services are providers more comfortable offering to adolescent girls and young women, and why? Why do you think some staff feel confident in counseling adolescents for certain family planning methods over others? Are there any gaps in training for family planning providers in offering these services to adolescents?
- Are there any issues from the facility side in providing these services consistently? *Potential probes: method stockouts, lack of time, clinic closures, etc.*
- How would you describe the support from parents, peers, or partners for adolescents in using family planning more generally? And how does that differ between parents, peers, and partners? How does that vary also by specific family planning method?
- What problems have you and your patients experienced in providing and using various family planning methods?
- Is there stigma surrounding use of family planning methods amongst adolescent girls and young women? *Potential probes: for example, that adolescent girls and young women should not be having sex before marriage thus should not need family planning, or that using family planning means a young woman is sinful*

**Domain 3: Long-acting ART use**

“We are now going to move onto the third section of our discussion. Long-acting antiretrovirals (ARVs), such as injectable ARV therapy (ART), may enhance viral suppression in some persons and are being investigated in substitution of currently available oral ARVs. These will be monthly injections that will allow users to avoid daily oral antiretroviral therapy. Long-acting ARVs are also under study for prevention, but for our time today we will focus on long-acting ART only.”

*Program or intervention- general of who, acceptability*

- Tell us first what you know or have heard about injectable ART?
- Do you believe that this form of HIV treatment will be an acceptable alternative to oral treatment? Tell us more in terms of how complicated or difficult its use will be or how comfortable patients and providers will be with injectable ART?
- Do you understand the risks, benefits, and mechanisms of injectable ART? Could you please comment on your understanding of these topics and provide further details?
- Do you think people in the 15-24 year old age group who are currently using ART would prefer to use injectable ART rather than oral ART? Why or why not? How might these factors differ between adolescents vs. adults?
- What types of patients do you think would benefit the most from injectable ART?
- How can we reach the largest number of adolescents & young women living with HIV to offer them injectable ART as an HIV treatment option?

*Benefits*

- What potential benefits do you foresee with injectable ART use compared to oral ART? For example, do you think patients would have better adherence using injectable ART compared with oral ART? Why or why not?

*Anticipated challenges or drawbacks*

- What challenges or drawback do you anticipate with providing injectable ART? What challenges do you anticipate from the patient side, from the provider/facility side?

🡪*Potential probes:*

- *Fear of jabs*
- *Handling side effects, drug-drug interactions*
- *Transportation costs, travel time*
- *School or holiday schedules*
- *Provider training/attitudes*
- *Facility logistics, supply chain, stockouts*
- Why would some individuals refuse injectable ART as a form of treatment? For example, do you think they will be concerned about whether it is effective or about the fact that it is still being studied?
- Is there anything providers or clinics can do to improve the patients’ experience with injectable ART to make this treatment successful for them?
- What are some potential factors that will make it simpler to implement injectable ART usage in your facility?

*Overlap, convergence and divergence with long-acting ART and FP*

“For the next section, I want to ask about your thoughts of where there might be overlap or synergies between long-acting ART and long-acting family planning. When we say long-acting family planning, we IUDs, implant, and also injectable DMPA. We recognize that usually in the family planning world, DMPA is not considered long-acting. But since the first long-acting ART will be injectable, we want to understand how there might be synergy between injectable ART and DMPA.”

- Where do you think there might benefits of providing and using both long-acting ART & long-acting family planning?
- Where might there be drawbacks or difficulties with providing long-acting ART with long-acting family planning?

**Domain 4: Health systems readiness**

“Now we will talk about the 4^th^ and last section of our discussion today. The focus now is on health systems readiness for long-acting ART. I will ask first about readiness for your clinic and later from the larger perspective at the national level.”

*Feasibility (defined as the extent to which a new treatment, or an innovation, can be successfully used or carried out within a given agency or setting)*

- Do you think your clinic is ready to provide injectable ART?

*🡪 Potential probes:*

- Do you think providers will be able to counsel patients and respond to questions from patients’ and their parents about injectable ART?
- Who would be giving the injectable ART in your clinic? For example, who usually gives injections in your clinics? What types of training do you think these persons might need above and beyond training they already receive?
- Do you have the supplies needed, for example, the needles & syringes?
- Do you think your clinic has the capacity to see patients monthly for injections, compared to how frequently they are being seen while on oral ART?
- Do you think you can properly monitor patients using injectable ART?
- For each question above, if the answer is “no”, what will be needed for your clinic to provide injectable ART?
- Is there anything else you think you will need for your clinic to be ready to provide injectable ART?
- What additional complications or issues should your facility consider in providing injectable ART for adolescents & young women in your clinic? Is there anything you are worried about in providing injectable ART for adolescents & young women in your clinic?
- What do you think the study team could do to be helpful to you in providing injectable ART for adolescents & young women in your clinic?

*Fidelity (defined as the degree to which an intervention was implemented as it was prescribed in the original protocol or as it was intended by the program developers. The literature identifies five implementation fidelity dimensions including adherence, quality of delivery, program component differentiation, exposure to the intervention, and participant responsiveness or involvement)*

- How do you think we can best monitor the process of providing injectable ART to adolescents & young women in your clinic? For example, how can we be sure patients are being offered this option for ART? How can we be sure it is being provided correctly & patients are being monitored correctly? How can we be sure patients are feeling comfortable while using injectable ART?
- How do you think we can continually improve the provision of injectable ART to make sure it is improving care for your patients?

*Recipients*

- How can your clinic best get patients ready to learn about injectable ART?
- How should patients be best counseled for injectable ART?

*External environment*

- What things need to happen outside of your facility to prepare for the roll-out of injectable ART within your clinic and others?

*🡪* Potential probes:

- What guidance do you need from AMPATH program or MTRH leadership?
- What guidance do you need from Kenya MOH or NASCOP? Any from WHO or other international bodies?
- What guidance or support do you need from the county or subcounty health departments or CASCOs?
- What other approvals or guidance would you want in place before offering injectable ART in your clinic?

*Implementation and sustainability*

- What factors do you think are key for successful implementation of injectable ART?
- What factors do you think are key considerations for sustainability of offering injectable ART?

**“**We have come to the end of our interview. Does anyone have any additional comments relating to the topic we have been discussing?

Thank you very much for your time and responses to the questions.”
