# Supplementary material for: Addressing barriers and facilitators to family planning uptake among adolescent girls and young women in the anticipated context of long-acting antiretroviral therapy (LA ART)
Source: PLOS Glob Public Health. 2026 Nov 10;6(11):e0007546. doi: 10.1371/journal.pgph.0007546 (PMC13750552; doi:10.1371/journal.pgph.0007546)
Supplement: Supplementary file 4 [file pgph.0007546.s004.docx]

**Focus group discussion guide for advocates for people living with HIV**

“My name is _______________. Thank you all for your willingness to be a part of this discussion. I am part of the Kuwa Free study. The main goal of this discussion is to better understand your perspectives as advocates for people living with HIV, and the feelings and choices of the people who you advocate for, regarding long-acting antiretroviral therapy (ART), as well as family planning.

Please know there are no right or wrong answers and we welcome every opinion about the topics we will discuss, so feel free to share your thoughts, opinions and views openly. If during our discussion, there are issues or concerns that you would like to talk about, feel free to bring them up, even if I don’t ask about them. If you have specific questions during the interview, I will take note of them and answer them directly after the interview. If I cannot answer them, I can refer you to someone who may be able to help. Before we start, can you confirm that you have already provided written informed consent to take part in this discussion? [Wait for oral confirmation to begin].

Despite the audio recorder, I want to remind you all that what we discuss here will be kept confidential, and that we will not share your personal information or responses with anyone outside of the study. You will not be judged by your responses. This discussion will take approximately 60 minutes.

Does anyone have any questions before we get started? Do you all agree to have the discussion audio recorded?”

*Interviewer: switches recorder on*

“Okay, let’s begin.”

***(Interviewer: Ensure the interviewees’ demographics are recorded and assign IDs. Provide date, group being interviewed, time, study ID of all participants being interviewed.)***

**Domain 1: Advocacy for HIV differing between adults vs. adolescents**

“To start this up, I will ask about your experiences with advocacy for people living with HIV in the first section.”

- What is your role in advocating for people living with HIV? What has your experience been like to date?
- If you have advocacy activities separately for adolescents, can you comment on how those are different for adolescents or adults? *Potential probes:*
  - Different needs for adolescents vs adults living with HIV
  - Different challenges/concerns of adolescents vs adults living with HIV
  - Nuances to disclosure, stigma
  - Issues with adherence, including due to residential schools

**Domain 2: Advocacy around Family Planning**

“Thank you all for such a good discussion so far. I am now going to transition us to the second section that focuses on advocacy efforts around family planning. Some of you may have experience with advocacy for family planning & some not, that is ok, you can all speak to any experience you have.”

- What is your role in advocating for family planning, either for people living with HIV or not specifically for people living with HIV? What has your experience been like to date?
- If you have advocacy activities separately for family planning for adolescents, can you comment on how those are different for adolescents or adults? *Potential probes:*
  - Different FP needs/challenges/concerns for adolescents vs adults living with HIV
  - Nuances to disclosure, stigma around HIV & FP (*e.g. relationship with/role of parents, partners, teachers, that adolescent girls and young women should not be having sex before marriage thus should not need family planning, or that using family planning means a young woman is sinful*)
  - Problems using particular FP methods for adolescents vs adults (*e.g. issues with adherence to FP methods like condoms or pills, including due to residential schools*)
  - Myths around particular FP methods like IUCDs/implants for adolescents vs adults
  - Different FP discussions/needs for male vs. female adolescents
- If you have advocacy activities for family planning separately for people living with HIV compared to those without, can you comment on how those are different? *Potential probes:*
  - Different FP needs/challenges/concerns for those living with vs living without HIV
  - Nuances to disclosure, stigma around FP for those living with HIV
  - Problems using particular FP methods, e.g. Issues with adherence to FP methods like condoms or pills, for those living with vs living without HIV
  - Myths around particular FP methods like IUCDs/implants for those living with vs living without HIV
- What problems have you faced in advocating for family planning that is specific to adolescents girls or young women? *Potential probes:*
  - misinformation in community or from parents
  - belief that adolescents are not or should not be having sex
  - conflicting religious beliefs around family planning use
  - provider bias against offering long-acting family planning methods to adolescents
  - gaps in training for providers or issues at facilities offering FP services to adolescents
- How would you describe the support in the community or from parents, peers, or partners for adolescents in using family planning more generally? *Potential probes:*
  - differences between parents, peers, and partners
  - differences around specific family planning methods
  - problems using various family planning methods

**Domain 3: Long-acting ART use**

“We are now going to move onto the third section of our discussion. Long-acting antiretrovirals (ARVs), such as injectable ARV therapy (ART), may enhance viral suppression in some persons and are being investigated in substitution of currently available oral ARVs. These will be monthly injections that will allow users to avoid daily oral antiretroviral therapy. Long-acting ARVs are also under study for prevention, but for our time today we will focus on long-acting ART only.”

*Program or intervention- general of who, acceptability*

- Tell us first what you know or have heard about injectable ART? Where did you hear about this? What has the discussion been in the communities of people living with HIV who you advocate for?
- Do you believe that this form of HIV treatment will be an acceptable alternative to oral treatment for the people you advocate for? Tell us more in terms of how complicated or difficult its use will be or how comfortable people living with HIV will be with injectable ART, what types of people would benefit the most from injectable ART, etc.
- Do you think people in the 15-24 year old age group who are currently using ART would prefer to use injectable ART rather than oral ART? Why or why not? How might these factors differ between adolescents vs. adults?
- How can we reach the largest number of adolescents & young women living with HIV to offer them injectable ART as an HIV treatment option? What role can advocates such as yourselves play in reaching them?

*Benefits*

- What potential benefits do you foresee with injectable ART use compared to oral ART for the people you advocate for? For example, do you think they would have better adherence using injectable ART compared with oral ART? Why or why not?

*Anticipated challenges or drawbacks*

- What challenges or drawbacks do you anticipate with injectable ART for the people you advocate for? What challenges do you anticipate with injectable ART from the provider/facility side? *Potential probes:*
- *Fear of new or “experimental” medications or changing medications*
- *Fear of jabs*
- *Handling side effects, drug-drug interactions*
- *Transportation costs, travel time*
- *School or holiday schedules*
- *Provider training/attitudes*
- *Facility logistics, supply chain, stockouts*
- Whose concerns need to be taken into consideration when creating programs or policies around implementation of long-acting ART?
- Why would some individuals refuse injectable ART as a form of treatment? For example, do you think they will be concerned about whether it is effective or about the fact that it is still being studied? (*Can ask if not already covered with previous probes)*
- Is there anything providers or clinics can do to improve the experience of people living with HIV with injectable ART to make this treatment successful for them?
- What are some potential factors that will make it simpler or more beneficial to implement injectable ART usage for people you advocate for?
- Any other barriers to implementation you can anticipate?

“For the next section, I want to ask about your thoughts of whether it might be beneficial to provide both long-acting ART and long-acting family planning for the people you advocate for. When we say long-acting family planning, we mean IUCDs, implants, and also injectable Depo.”

- Do you think there might be benefits to using both long-acting ART & long-acting family planning for the people you advocate for?
- Where might there be drawbacks or difficulties with using both long-acting ART and long-acting family planning?

**Domain 4: Health systems readiness**

“Now we will talk about the 4^th^ and last section of our discussion today. The focus now is on health systems readiness for implementing long-acting ART.”

*Feasibility (defined as the extent to which a new treatment, or an innovation, can be successfully used or carried out within a given agency or setting)*

- Do you think the programs where people you advocate for receive HIV care, the county health systems, or the MOH in Kenya are ready to provide injectable ART?

*Potential probes:*

- What messaging do people living with HIV need to hear about injectable ART before its roll-out?
- What training do advocates need about injectable ART to assist in advocating for people living with HIV during the rollout and for long-term success of the program?
- What systems need to be in place at the national, county, and program levels for the rollout of injectable ART?
- What systems need to be in place to support people living with HIV to successfully use injectable ART?
- What role can advocates play in the success of these systems?
- What additional complications or issues should we consider in providing injectable ART for adolescents & young women? Is there anything you are worried about for adolescents & young women receiving injectable ART?
- What do you think the study team could do to be helpful to your groups in advocating for injectable ART for adolescents & young women?

*Fidelity (defined as the degree to which an intervention was implemented as it was prescribed in the original protocol or as it was intended by the program developers. The literature identifies five implementation fidelity dimensions including adherence, quality of delivery, program component differentiation, exposure to the intervention, and participant responsiveness or involvement)*

- How do you think we can best monitor the process of providing injectable ART to adolescents & young women? For example, how can we be sure people living with HIV are being offered this option for ART? How can we be sure patients are feeling comfortable while using injectable ART? What stakeholders, including which advocacy organizations, do we need to make sure receive information from our study?
- How do you think we can continually improve the provision of injectable ART to make sure it is improving care for people living with HIV?

*Recipients*

- Other than patients and providers, who else needs to be prepared for rollout of injectable ART? For example, which advocacy organizations should be involved? Which other people or organizations that you work with? E.g, CASCO or other county level managers, implementation partners, community health workers, etc?

*External environment*

- What things need to happen in the community to prepare for the roll-out of injectable ART? *Potential probes:*
  - What types of advocacy activities will be needed?
  - Which organizations can best provide guidance & support for these activities? For example AMPATH, MTRH, Kenya MOH, NASCOP, WHO etc?
  - What types of guidance or support do you need from these organizations?

*Implementation and sustainability*

- What factors do you think are key for successful uptake & long-term sustainability of injectable ART?

*Potential probes:*

- Messaging- what messaging do people with HIV need for this to be a success? And how best to do that?
- Support- whose support in the community do we need to make this successful for people living with HIV?

**“**We have come to the end of our interview. Does anyone have any additional comments relating to the topic we have been discussing?

Thank you very much for your time and responses to the questions.”
