# Supplementary material for: Addressing barriers and facilitators to family planning uptake among adolescent girls and young women in the anticipated context of long-acting antiretroviral therapy (LA ART)
Source: PLOS Glob Public Health. 2026 Nov 10;6(11):e0007546. doi: 10.1371/journal.pgph.0007546 (PMC13750552; doi:10.1371/journal.pgph.0007546)
Supplement: Supplementary file 5 [file pgph.0007546.s005.docx]

**Focus group discussion guide for policymakers**

“My name is _______________. Thank you all for your willingness to be a part of this discussion. I am part of the Kuwa Free study. The main goal of this discussion is to better understand your perspectives regarding availability and implementation of long-acting antiretroviral therapy (ART) as well as integration of family planning into these care programs.

Please know there are no right or wrong answers and we welcome every opinion about the topics we will discuss, so feel free to share your thoughts, opinions and views openly. If during our discussion, there are issues or concerns that you would like to talk about, feel free to bring them up, even if I don’t ask about them. If you have specific questions during the interview, I will take note of them and answer them directly after the interview. If I cannot answer them, I can refer you to someone who may be able to help. Before we start, can you confirm that you have already provided written informed consent to take part in this discussion? [Wait for oral confirmation to begin].

Despite the audio recorder, I want to remind you all that what we discuss here will be kept confidential, and that we will not share your personal information or responses with anyone outside of the study. You will not be judged by your responses. This discussion will take approximately 60 minutes.

Does anyone have any questions before we get started? Do you all agree to have the discussion audio recorded?”

*Interviewer: switches recorder on*

“Okay, let’s begin.”

***(Interviewer: Ensure the interviewees’ demographics are recorded and assign IDs. Provide date, group being interviewed, time, study ID of all participants being interviewed.)***

**Domain 1: Policy-making for HIV and adolescent care**

“To start this up, I will ask about your experiences and roles within policymaking around HIV care and adolescent care in the first section.”

- What has your experience been in program development or policymaking around people living with HIV?
- How do you think policymaking or program development around the care of adolescents is different than for adults? In your opinion, what are some differences in how policy or program priorities are decided for adolescents vs. adults living with HIV? *Potential probes:*
  - Regulatory issues around adolescents vs adults
  - Implementation & scale-up concerns
  - Nuances to disclosure, stigma, parental involvement, adherence
  - Role/input of educational leadership/institutions
  - Religious or political perspectives/objections
  - Other probes?

**Domain 2: Policy-making for Long-acting ART**

“As you may be aware, long-acting ART via monthly or every two monthly injections are now approved for HIV treatment in other settings and will be soon approved in Kenya. Additionally, other oral tabs, injections, or implants that can be taken at monthly, six-monthly, or longer intervals are under development. For the next section, we would like to hear about policy-making and potential barriers and facilitators to implementation of long-acting ART, and specifically the injectable cabotegravir and rilpivirine that are on the horizon for use in Kenya.”

***Facilitators to implementation of long-acting ART***

- Tell us first what you know or have heard about injectable ART? Do you believe that this form of HIV treatment will be an acceptable alternative to oral treatment for policymakers, providers, and patients?
- What potential benefits do you foresee with injectable ART use compared to oral ART? What types of patients do you think would benefit the most from injectable ART?
- How can we reach the largest number of adolescents & young women living with HIV to offer them injectable ART as an HIV treatment option?
- From your experiences discussing and deciding policies around the care of adolescents with HIV, what specific facilitators exist that will be helpful in the process of implementation and scale up of long-acting ART for adolescents and young adults? Are these different from those facilitators that need to be considered for adults?

*Potential probes: Describe any facilitators associated with:*

- ***individual-***
- adherence
- alcohol or other drug use
- lack of food or other related issues
- acquired HIV drug resistance
- drug-drug interactions
- ***interpersonal-***
- disclosure
- stigma
- lack of privacy
- parent/family/partner support
- peer support
- provider attitudes
- ***organizational-***
- provider training
- provider strikes
- supply chain
- drug stock outs
- transportation to facilities
- school and holiday schedules
- travel time, or long waits at clinics
- ***societal/cultural norms-***
- adolescent autonomy
- cultural norms for adolescents taking lead for their health
- gender norms around ART use
- ***policy/structural-***
- legal status of adolescents
- discrimination against those living with HIV
- unemployment and scarcity of financial resources, poverty

***Barriers to implementation of long-acting ART***

- From your experiences discussing and deciding policies around HIV care, what barriers or challenges do you think need to be taken into consideration in policy-making for implementation and scale up of long-acting ART for adolescents and young adults? Are these different from those barriers that need to be considered for adults?

*Potential probes:* Describe any barriers associated with:

- ***individual-***
- adherence
- alcohol or other drug use
- lack of food or other related issues
- acquired HIV drug resistance
- drug-drug interactions
- ***interpersonal-***
- disclosure
- stigma
- lack of privacy
- parent/family/partner support
- peer support
- provider attitudes
- ***organizational-***
- provider training
- provider strikes
- supply chain
- drug stock outs
- transportation to facilities
- school and holiday schedules
- travel time, or long waits at clinics
- ***societal/cultural norms-***
- adolescent autonomy
- cultural norms for adolescents taking lead for their health
- gender norms around ART use
- ***policy/structural-***
- legal status of adolescents
- discrimination against those living with HIV
- unemployment and scarcity of financial resources, poverty
- Whose concerns need to be taken into consideration when creating policies around implementation of long-acting ART?
- What kinds of policies would make this implementation more difficult?
- Any other barriers to implementation you can anticipate?

**Domain 2: Policy-making for Family Planning**

“Thank you all for such a good discussion so far. I am now going to transition us to the second section that focuses on policy-making around family planning and where there might be overlap or synergies between long-acting ART and long-acting family planning. As part of this project we are aiming to see how we can integrate long-acting family planning into long-acting ART use. When we say long-acting family planning, we mean IUDs, implant, and also injectable DMPA. We recognize that usually in the family planning world, DMPA is not considered long-acting. But since the first long-acting ART will be injectable, we want to understand how there might be synergy between injectable ART and DMPA.”

- Can you describe any differences in considerations for policy-making around family planning for adolescents girls vs. young women vs. older women?
- What challenges exist in creating policies around family planning? Anything specific to adolescents girls or young women? *Potential probes:*
  - provider challenges like lack of time to counsel adequately, gaps intraining for providing particular methods, bias against offering long-acting methods to adolescents
  - facility issues like method stockouts, clinic closures, etc.
  - community challenges like stigma, religious or cultural beliefs, or misinformation about family planning
  - the role of parents or other adults in the decision-making for adolescents
  - Different FP needs for adolescents vs adults living with HIV
  - Different challenges/concerns around FP for adolescents vs adults living with HIV
  - Nuances to disclosure, stigma around HIV & FP (*e.g. relationship with/role of parents, partners, teachers, that adolescent girls and young women should not be having sex before marriage thus should not need family planning, or that using family planning means a young woman is sinful*)
  - Issues with adherence to FP methods like condoms or pills
  - Myths around particular FP methods like IUCDs/implants for adolescents vs adults
  - Problems using particular FP methods for adolescents vs adults
  - Different FP discussions/needs for male vs. female adolescents
- What type of policy-making around family planning services do we need to take into consideration for integration of family planning into long-acting ART?
- What facilitators do you see to integration of family planning into long-acting ART services? *Potential probes:*
  - what kinds of existing policies will be helpful to this integration
  - what future policies could be created to help with this integration
- What barriers do you see to integration of family planning into long-acting ART services? *Potential probes:*
  - what kinds of existing policies will make this integration challenging?
  - what potential future policies could make this integration challenging?

**Domain 3: Health systems readiness**

“Now we will talk about the last section of our discussion today. The focus now is on health systems readiness for long-acting ART.”

*Feasibility (defined as the extent to which a new treatment, or an innovation, can be successfully used or carried out within a given agency or setting)*

- Do you think your program, county, or country is ready to provide injectable ART?

*Potential probes:*

- What messaging do patients need to hear about injectable ART before its roll-out? How can policy-makers facilitate this messaging?
- What training do providers need about injectable ART before its rollout? How can policy-makers facilitate this training?
- What policy and systems need to be in place at national, county, and program level for rollout of injectable ART?
- What policies and systems need to be in place to properly monitor patients using injectable ART?
- Can you comment on any feasibility challenges in dosing injectable ART every 2 months instead of every month?
- What data or information from our study will be helpful in setting appropriate policies for the implementation of injectable ART for adolescents & young women?
- What additional concerns or issues should be considered in creating policy around injectable ART for adolescents & young women? Is there anything you are worried about in providing injectable ART for adolescents & young women?
- What else do you think the study team could do to be helpful to you in creating policies around injectable ART for adolescents & young women?

*Fidelity (defined as the degree to which an intervention was implemented as it was prescribed in the original protocol or as it was intended by the program developers. The literature identifies five implementation fidelity dimensions including adherence, quality of delivery, program component differentiation, exposure to the intervention, and participant responsiveness or involvement)*

- How do you think we can best monitor the process of providing injectable ART to adolescents & young women and what types of policies will need to be in place for this long-term? For example, how can we be sure patients are being offered this option for ART? How can we be sure it is being provided correctly & patients are being monitored correctly? How can we be sure patients are feeling comfortable while using injectable ART? What stakeholders do we need to engage with data derived from our study?
- How do you think we can continually improve the provision of injectable ART to make sure it is improving care for people living with HIV? What kinds of policies can be helpful in this continual improvement process?

*Recipients*

- Other than patients and providers, who else needs to be prepared for rollout of injectable ART? E.g, CASCO, other county level managers, implementation partners?

*External environment*

- What things need to happen outside of a facility to prepare for the roll-out of injectable ART?

Potential probes:

- What types of policies need to be in place before the rollout of injectable ART?
- Who should these policies come from? For example, AMPATH program, MTRH leadership, Kenya MOH, NASCOP, WHO or other international bodies, county or subcounty health departments or CASCOs?
- What other approvals or guidance need to be in place before offering injectable ART in the clinics?

*Implementation and sustainability*

- What factors do you think are key for successful implementation of injectable ART? How can policies be created to facilitate this successful implementation?
- What factors do you think are key considerations for sustainability of offering injectable ART? How can policies be created to facilitate this successful implementation?

**“**We have come to the end of our interview. Does anyone have any additional comments relating to the topic we have been discussing?

Thank you very much for your time and responses to the questions.”
